# Three-arm randomized controlled trial evaluating the effectiveness of the self-assembling peptide P11-4 in the remineralization of enamel carious white spot lesions: a study protocol

**DOI:** 10.3389/froh.2026.1932140

**Published:** 2026-10-07

**Authors:** Anna Turska-Szybka, Julia Sazonowa, Dorota Olczak-Kowalczyk, Elżbieta Wójcik-Gront, Giusy Rita Maria La Rosa

**Affiliations:** 1 Department of Paediatric Dentistry, Medical University of Warsaw, Warsaw, Poland; 2 Department of Paediatric Dentistry, University Dental Center, Warsaw, Poland; 3 Department of Biometrics, Faculty of Agriculture and Ecology, Warsaw University of Life Sciences, Warsaw, Poland; 4 Clinical and Experimental Medicine, University of Catania, Catania, Italy

**Keywords:** fluoride varnish, quantitative light-induced fluorescence, randomiced clinical trial, remineralization, self-assembling peptide

## Abstract

**Background:**

Enamel carious white spot lesions (WSLs) represent early-stage demineralization that may progress to cavitated lesions requiring invasive treatment. The self-assembling peptide P11-4 (SAP P11-4) is a biomimetic agent designed to promote enamel remineralization by forming a biomatrix that attracts calcium and phosphate ions and supports hydroxyapatite formation. Although evidence supports its efficacy, the optimal application protocol remains unclear, and no clinical trial has directly compared single vs. repeated SAP P11-4 application.

**Objective:**

To compare the remineralization efficacy of repeated vs. single SAP P11-4 application in adolescents with enamel carious WSLs at the prespecified 6-month primary time point, while also evaluating both SAP P11-4 regimens against fluoride varnish alone over 12 months.

**Methods:**

This three-arm, partially blinded, randomized controlled trial will enroll participants aged >10 and <18 years with at least two active enamel carious WSLs on smooth surfaces of permanent teeth. Participants will be randomly allocated 1:1:1 to Group A (single SAP P11-4 application + FV), Group B (SAP P11-4 applications at baseline and 3 months + FV), or Group C (FV alone). The planned sample size is 75 participants (25 per group). The primary outcome is the change in the QLF-derived lesion volume parameter (Δ*Q*) from baseline to 6 months at the lesion level, comparing repeated vs. single SAP P11-4 application. Secondary outcomes include longitudinal changes in QLF measures, fluorescence loss (Δ*F*), clinical lesion activity assessed using ICDAS II, ICDAS-LAA, and LAA criteria, periodontal parameters, and patient-reported tolerability assessed by visual analogue scale and adverse event recording. QLF assessments will be performed by a blinded investigator. Linear mixed-effects models will account for repeated measurements and multiple lesions nested within patients.

**Results:**

The study was approved by the Bioethics Committee of the Medical University of Warsaw (KB/1/2026, 12 January 2026). Recruitment is planned to commence in February 2026, with study completion expected in July 2027.

**Conclusions:**

This trial will provide evidence on the comparative efficacy of single vs. repeated SAP P11-4 application and inform an evidence-based clinical protocol for managing enamel WSLs in adolescents.

**Clinical Trial Registration:**

https://clinicaltrial.gov/, identifier NCT07664046.

## 1 Introduction

A white spot lesion (WSL) presents as a chalky white opacity on a smooth tooth surface, caused by subsurface enamel demineralization (1). If left untreated, they may progress to cavitated lesions requiring invasive treatment. Various therapeutic approaches have been proposed to prevent and manage white spot lesions, such as topical fluoride, CPP-ACP-based products, and resin infiltration. Nevertheless, many of these methods mainly promote surface-level remineralization and may be insufficient to effectively reverse deeper subsurface lesions (2).

A newer option in preventive dentistry is the self-assembling peptide P11-4 (SAP P11-4), which is designed to support the tooth's natural enamel remineralization (3, 4).

The self-assembling peptide P11-4 constitutes a biomimetic approach for the treatment of early carious lesions. In this formulation, the peptide is present as a free monomer and in a liquid form. It enables deep remineralization of early carious lesions such as white spot lesions through enamel regeneration. The peptide forms a biomatrix with high affinity for hydroxyapatite. The biomatrix attracts calcium and phosphate ions derived from saliva, resulting in the formation of new hydroxyapatite crystals and enamel regeneration (5, 6).

Both *in vitro* and clinical studies have demonstrated that SAP P11-4 significantly inhibits the progression of early carious lesions and induces their regression, reducing lesion size compared with fluoride agents alone (7–11); furthermore, its efficacy is enhanced when combined with fluoride varnish (FV) (10). However, existing evidence is limited by short follow-up periods and longer-term studies are warranted (12). In addition, the optimal application protocol remains to be established: whereas the manufacturer recommends a single application, Güven et al. (5) observed that the remineralization benefit of a single SAP P11-4 application was evident at three months but was not sustained relative to fluoride varnish with tricalcium phosphate at six months, suggesting that repeated application may be needed to maintain efficacy over time. According to Shaalan et al. (13), further controlled clinical trials are needed to assess the effect of repeated applications of the combination of SAP P11-4 and FV over a prolonged period of time to verify its effectiveness and any possible adverse effects.

These prior studies vary considerably in design: most (7–9, 12, 13) are randomized controlled trials testing a single application per treatment arm, while others (10, 11) are *in vitro* investigations, and only one (5) is a non-randomized *in vivo* comparison. Critically, none of these studies has directly compared single vs. repeated application of SAP P11-4 within the same randomized design, and follow-up periods rarely extend beyond 12 months, underscoring the need for the present trial.

Conventional caries detection methods, such as visual and tactile inspection, may fail to identify the earliest lesions, particularly on smooth surfaces close to the gingival margin. Over the past years, light-based techniques have significantly advanced caries detection and evaluation. Quantitative light-induced fluorescence (QLF) is one of these methods (14); QLF enables quantitative assessment of demineralized enamel by measuring fluorescence loss in the lesion area relative to sound enamel, which reflects the degree of mineral loss (15). It facilitates the detection of early non-cavitated caries lesions and may support the estimation of lesion depth (16). Compared with conventional clinical and radiographic examinations, QLF may provide a more sensitive, simple, and non-invasive approach for detecting and monitoring early caries lesions (17).

To date, no clinical study has directly compared a single application of SAP P11-4 with repeated application in the management of enamel white spot lesions, representing a critical gap in the evidence base. The present randomized controlled trial (RCT) is therefore designed to address this question.

### 1.1 Objectives and hypothesis

The primary objective is to evaluate the remineralization efficacy of repeated vs. single SAP P11-4 application in the management of enamel carious white spot lesions, assessed by the change in the QLF-derived lesion volume parameter (Δ*Q*) from baseline to 6 months.

The secondary objective is to evaluate the remineralization efficacy of the SAP P11-4 in the management of enamel carious white spot lesions across follow-up visits, assessed via QLF-derived parameters (Δ*F*, Δ*Q*, and lesion area) and clinical criteria, by comparing:

1. Single application of SAP P11-4 + fluoride varnish;
2. Repeated application of SAP P11-4 + fluoride varnish;
3. Fluoride varnish alone.

Null hypotheses (H0):

The null hypothesis (H0) is that there is no difference in the change in Δ*Q* from baseline to 6 months between the repeated-application and single-application SAP P11-4 groups.

## 2 Methods

### 2.1 Study design

The study is designed as a three-arm randomized controlled trial.

After meeting the inclusion and exclusion criteria and providing written informed consent, participants will be randomly allocated to one of three groups in a 1:1:1 ratio:

1. **Test group A (Group A)**—single application of SAP P11-4 [Curodont Repair (CR), Switzerland]; and application of fluoride varnish (Duraphat Fluoride Varnish Colgate, 22,600 ppm F).
2. **Test group B (Group B)**—Repeated application of SAP P11-4 [Curodont Repair (CR), Switzerland] and fluoride varnish (Duraphat Fluoride Varnish Colgate, 22,600 ppm F). The three-month interval for the second SAP P11-4 application in Group B was selected based on evidence that the remineralization benefit of a single application, although evident at three months, may not be sustained relative to comparator treatments thereafter (5). This timing also coincides with the first scheduled follow-up visit (V2), avoiding an additional dedicated appointment for participants
3. **Control group (Group C)**—application of fluoride varnish Duraphat Fluoride Varnish Colgate, 22,600 ppm F.

The SAP P11-4 application for the study will be purchased by the Department of Paediatric Dentistry, WUM, as part of the Department's internal research project. Participants will not incur treatment costs. Each visit is expected to last approximately 30 min (the application procedure for both SAP P11-4 and fluoride varnish takes approximately 5–10 min; the patient interview and clinical examination approximately 20 min). Permissible deviations from the scheduled visit time should not exceed 2 weeks.

### 2.2 Study population

Patients aged >10 years and <18 years, living in Poland, in whom at least two enamel carious white spot lesions are identified on smooth (labial/buccal) surfaces of permanent teeth, will be enrolled and randomly allocated to one of three groups. Participants will be recruited at the University Dental Center of the Medical University of Warsaw, Department of Paediatric Dentistry. The planned sample size is 75 participants, with 25 participants per group.

### 2.3 Sample size

Sample size was estimated using *a priori* power analysis assuming a two-sided significance level of *α* = 0.05 and statistical power of 80% (1 − *β* = 0.80). The primary outcome is the change in the QLF-derived Δ*Q* parameter from baseline to 6 months. Effect size assumptions were based on the best available external evidence, derived from Güven et al. (5), in which the between-group difference in Δ*Q* at 6 months was approximately 20 units (−21.92 ± 29.28 vs. −1.90 ± 16.27) (5). This estimate corresponds to the SAP P11-4-vs.-fluoride comparison and does not represent prior evidence for the expected difference between single and repeated SAP P11-4 application; it is treated as a pragmatic planning assumption, necessitated by the absence of directly relevant comparative data for the trial's primary comparison. A reliable ICC estimate was not available from directly comparable studies; therefore, no ICC-based design-effect correction was applied, and the sample-size calculation was performed at the patient level, consistent with the level of randomization. Under these assumptions, approximately 21 evaluable participants per group would provide 80% power. To allow for an anticipated attrition rate of approximately 15%, the recruitment target was set at 25 participants per group (total *N* = 75). Hierarchical dependence arising from repeated measurements and multiple lesions nested within patients will be addressed in the statistical analysis using linear mixed-effects models with random intercepts for patient and lesion nested within patient.

### 2.4 Participant eligibility criteria

#### 2.4.1 Inclusion criteria

To be eligible for enrolment in the study, a participant must meet the following criteria:

- Age >10 years and <18 years.
- Presence of at least 2 active enamel carious white spot lesions on a smooth tooth surface (labial/buccal surfaces of permanent teeth) based on International Caries Detection (ICDAS II) and Assessment System -Lesion Activity Assessment (ICDAS-LAA), and Lesion Activity Assessment (LAA criteria). The requirement of at least two active WSLs per participant reflects a study design choice aimed at increasing the number of lesion-level observations per patient, given that randomization is performed at the patient level and all eligible lesions in an enrolled participant receive the assigned treatment.
- Tooth eligible for treatment:

- Vital pulp,
- No loss of hard dental tissues within the investigated lesion (ICDAS II) code 1 or 2, LAA code 1, ICDAS-LAA>7;

- QLF score parameters consistent with demineralization without loss of tissue continuity- code 1 or 2),
- No history of trauma to the tooth.
- Systemically healthy patient.
- No contraindications to the use of fluoride preparations.
- No fixed orthodontic appliance.
- Written informed consent provided by the parent/legal guardian and, for participants aged >16 years, also by the participant.

#### 2.4.2 Exclusion criteria

Subjects will not be enrolled in the trial if any of the following exclusion criteria apply:

- Systemic diseases or chronic medication use due to chronic conditions.
- Viral/bacterial illness on the day of the visit, e.g., herpetic stomatitis, influenza, tonsillitis (temporary restriction).
- Pregnancy or breastfeeding.
- Allergy to any component of the SAP P11-4 preparation or the fluoride varnish.
- Professional fluoride prophylaxis performed <3 months ago.
- Presence of cavitation (loss of hard tissues) in the treated tooth—ICDAS II code ≥3, ICDAS-LAA<7, LAA code 0, 2–9.
- Carious WSL previously treated with the Icon resin infiltrant (methacrylate resin).
- Developmental enamel defects.
- Patient undergoing orthodontic treatment with fixed appliances.
- Inability to ensure regular follow-up visits.
- Lack of cooperation from the guardian or the patient.
- Refusal to participate in the study and/or absence of written consent.

### 2.5 Study endpoints

#### 2.5.1 Primary endpoints

Remineralization will be indicated by an increase in Δ*Q* values toward zero, accompanied by a reduction in lesion area. In the absence of an established MCID (minimal clinically important difference) for QLF-derived parameters in white spot lesions, clinical relevance will be interpreted by combining QLF changes with clinical lesion assessment.

The primary study endpoint is the quantitative change in the degree of demineralization measured using the QLF-derived lesion volume parameter (Δ*Q*) of carious white spot lesions (lesion level) between the baseline and the 6-month time point (prespecified primary time point), comparing repeated vs. single SAP P11-4 application:

- Change in the QLF parameter (Δ*Q*) between the baseline and the 6-month time point.
  Δ(ΔQ)=ΔQat6months−ΔQatthebaseline

Where:

- Δ*Q at the baseline*- WSL lesion volume at the baseline
- Δ*Q at 6 months—*WSL lesion volume at the 6-month follow-up
- Δ*(*Δ*Q)*—the change in WSL volume from the baseline to the 6-month time point

Lesion volume (Δ*Q*) is expressed as % × mm^2^ and combines lesion area and fluorescence loss. More negative Δ*Q* values indicate a larger or more severe lesion. Therefore, an increase in Δ*Q* toward zero, or a positive Δ(Δ*Q*), will indicate lesion improvement, whereas a decrease in Δ*Q*, or a negative Δ(Δ*Q*), will indicate lesion progression.

#### 2.5.2 Secondary endpoints

Secondary endpoints are assessed as the change from the baseline to each follow-up visit (3, 6, 9 and 12 months) and include:

- Change in QLF parameter's: fluorescence loss (Δ*F*), lesion area and Δ*Q*, from the baseline to each follow-up visit. For fluorescence loss (Δ*F*), values are expressed as percentage fluorescence loss (%), with more negative values indicating greater mineral loss. Therefore, an increase in Δ*F* toward zero, or a positive Δ(Δ*F*), will indicate lesion remineralization, whereas a decrease in Δ*F*, or a negative Δ(Δ*F*), will indicate lesion demineralization.
  Δ(ΔF)=ΔFatfollow−up−ΔFatbaseline

Where:

ΔF at baseline—WSL fluorescence loss at baseline
ΔF at follow-up—WSL fluorescence loss at the respective follow-up visit
Δ(ΔF)—the change in fluorescence loss from baseline to the respective follow-up visit

b. Change in ICDAS II, ICDAS-LAA, LAA to each follow-up visit.
c. Changes in oral hygiene/gingival status [Plaque Index (PLI), Gingival Index (GI), Bleeding on probing (BOP)] at each follow-up visit. These parameters are not expected to be directly modified by the remineralizing interventions; rather, they will be monitored as an indicator of the effect of the standardized oral hygiene instructions provided to all participants, as potential covariates in secondary/sensitivity analyses, and for safety/tolerability monitoring.
d. Patient-reported tolerability: Visual Analogue Scale (VAS) and occurrence of Adverse Events (AEs).

### 2.6 Study procedures

Visits will be scheduled in the morning hours, after breakfast and brushing their teeth with fluoride toothpaste. Patients will be asked not to eat for one hour before the visit due to saliva testing. The participant will be asked to report their current well-being and health status in order to exclude any temporary contraindications to the procedure.

At each visit (V1–V5), a standard dental examination will be performed including: intra-oral examination; saliva pH and unstimulated salivary flow rate (mL/min), PLI, GI, and BOP; assessment of calculus deposits; ICDAS II scoring, LAA, ICDAS-LAA, QLF imaging of treated lesions. Prior to ICDAS II, LAA, ICDAS-LAA and QLF imaging, plaque deposits will be removed using a toothbrush and fluoride-free paste. Relevant inclusion and exclusion criteria will be rechecked at each study visit (V0–V5) to identify any changes that could affect the participant's continued eligibility, safety, or ability to complete the study procedures.

Saliva variables will be used as potential covariates in secondary/sensitivity analyses.

#### 2.6.1 Recruitment period

The recruitment period will involve enrolling patients with at least two carious white spot lesions on smooth surfaces of permanent teeth. The presence of lesions will be confirmed based on a visual-tactile examination using the ICDAS II, ICDAS-LAA and LAA criteria. During the recruitment period, each patient and the patient's legal guardian will be asked about their willingness to participate in the study and will be informed about the study duration, potential adverse events, and the possibility to withdraw at any time. The guardian and patients aged over 16 years will also be informed that written informed consent must be provided before any study procedures are initiated. A preliminary eligibility assessment will be performed (inclusion and exclusion criteria).

Patients who require dental treatment during this period will receive appropriate treatment and preventive care. Each patient will receive standardized oral hygiene instructions. Patients will be instructed to brush their teeth at least twice daily, using a manual, sonic, or electric toothbrush and fluoride toothpaste containing 1450 ppm fluoride, for 2–3 min. In the case of a manual toothbrush, the patient will be instructed to use the modified Bass technique. The toothbrush should be positioned at a 45-degree angle to the gingival margin, with the bristles directed toward the gingival sulcus. Vibratory or circular movements should be performed in small sections, followed by a sweeping movement from the gingival margin toward the occlusal/incisal surface. In the case of an electric oscillating-rotating toothbrush, the patient will be instructed to place the brush head on each tooth surface separately, along the gingival margin, and allow the toothbrush to perform the cleaning movement without additional scrubbing. The brush head should be slowly moved from tooth to tooth, covering the buccal, lingual/palatal, and occlusal surfaces. In the case of a sonic toothbrush, the patient will be instructed to position the brush head at a slight angle toward the gingival margin and to move it slowly along the dental arch, allowing the sonic vibrations to clean the tooth surfaces. Patients will also be instructed and informed about the need for daily interdental cleaning, including the use of dental floss and/or interdental brushes, depending on individual interdental space anatomy.

As part of dietary counselling, each patient will receive an educational leaflet listing cariogenic and caries protective foods. Patients will be informed about the harmful effects of frequent consumption of cariogenic foods and drinks, especially those containing fermentable carbohydrates and sugars. They will also be instructed to brush their teeth after breakfast and before bedtime. After evening toothbrushing, patients will be advised not to eat any food and to drink only water.

The planned recruitment period is 6 months, from 1 February 2026 to 31 July 2026.

#### 2.6.2 Enrollment and randomization

Enrollment and randomization visit (V0) is planned two weeks prior to the baseline visit (V1). At this visit (V0), the following procedures are planned:

1. Assessment for eligible carious white spot lesions (at least two) on smooth surfaces of permanent teeth after removal of soft and/or hard deposits using a toothbrush and fluoride-free paste and/or scaler. Evaluation of white spot lesions based on visual-clinical assessment (ICDAS-II, ICDAS-LAA, LAA) and fluorescence loss of studied teeth (Δ*F*).
2. Verification of inclusion and exclusion criteria.
3. Provision of information to the parent/legal guardian and the participant regarding the planned visit schedule and overall study duration.
4. Obtaining written informed consent from the parent/legal guardian and the participant aged >16 years.

Randomization will be performed after completion of the clinical and visual-tactile examination and fluorescence loss of treated teeth and confirmation of at least two carious WSL on smooth surfaces of permanent teeth.

#### 2.6.3 Randomization

Randomization will be performed using computer-generated block randomization with blocks of 3 patients.

Allocation concealment will be ensured by the use of sequentially numbered, opaque, sealed envelopes, which will be opened only after a participant has been enrolled in the study. The person enrolling patients will not have access to the randomization sequence. The randomization sequence will be generated by a computer program and will be accessible only to an independent investigator who will not be involved in recruitment or in the analysis of the obtained results.

Patients will be randomly assigned to three groups labelled 1, 2, and 3 in a 1:1:1 ratio (using a random number generator/randomizer). Subsequently, using the randomizer, the assignment of groups 1/2/3 to Group A, Group B and Group C will be drawn.

#### 2.6.4 Blinding

The study will be blinded at the pre-randomization stage. Until randomization, the participant and investigators will not know the intended group allocation.

After randomization and allocation to one of the three treatment groups, participants will be informed of their assigned group (A/B/C). At this stage, the trial becomes partially blinded for two investigators, who will be aware of the assigned group and will be responsible for delivering the intervention, visual-tactile examination and documenting adverse events.

The third investigator will remain blinded throughout the study and will not have access to information on the participant's allocation. Only this investigator will be responsible for QLF assessments. The other two investigators will be blinded to the QLF measurement results.

To maintain blinding of the assessor, documentation related to treatment allocation, product application and clinical assessment records will be stored separately from QLF files completed by the blinded investigator. Any instance of inadvertent unblinding of the blinded investigator will be documented as a protocol deviation.

#### 2.6.5 The baseline visit (visit 1)

At the baseline visit (V1) the participant and/or the legal guardian will also be asked to reconfirm the inclusion and exclusion criteria to ensure that no relevant changes have occurred since the first visit (V0). The participant will be asked to complete an anonymous study questionnaire assessing the family's socioeconomic factors and containing questions on general health, oral hygiene practices and diet, dental attendance/previous dental visits, the participant's self-assessment of carious white spot lesions. Subsequently, the visit-specific procedures will be performed (Supplementary Tables S1–S3). The timing and schedule of study products application are illustrated in Figure 1.

**Figure 1:**
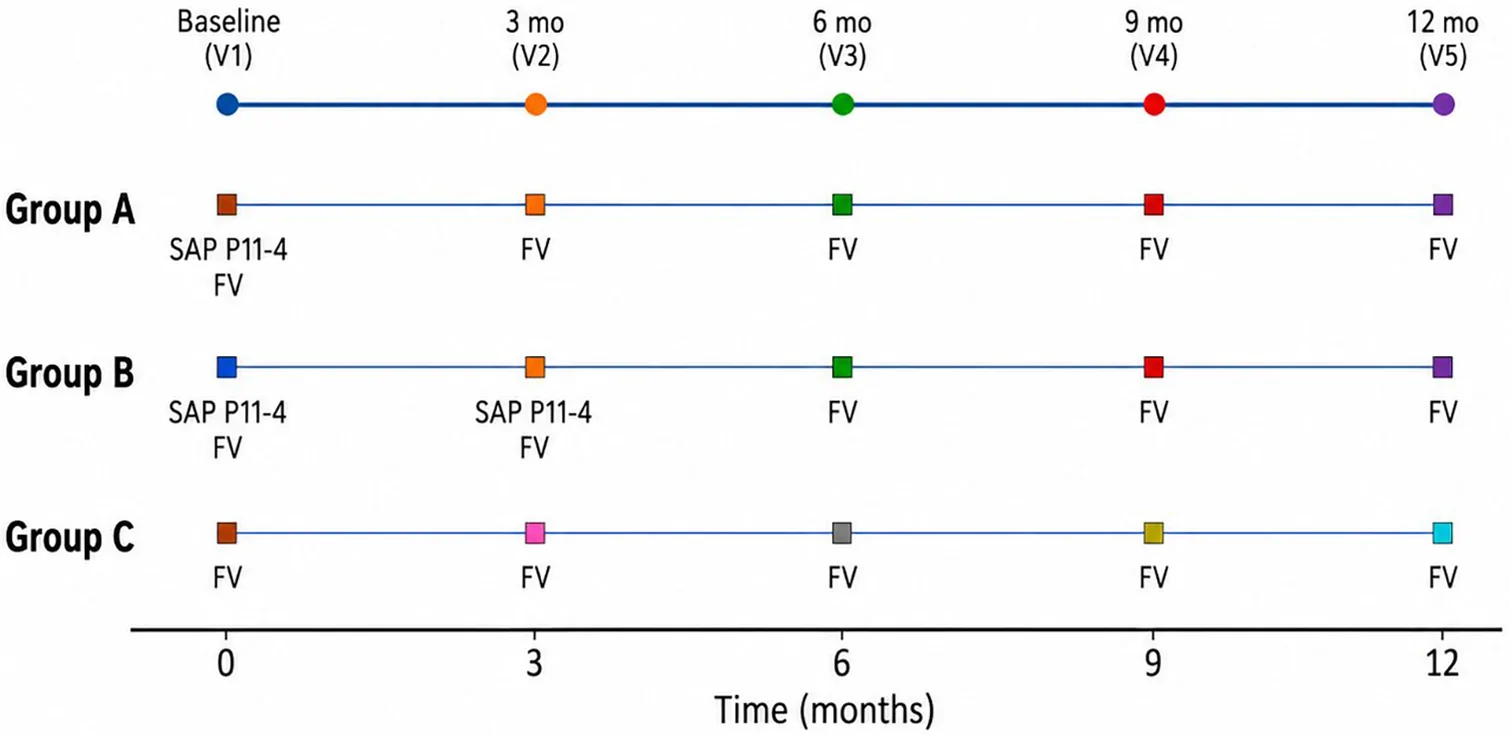
Study design schematic. Participants will attend five on-site visits: baseline (visit 1) and follow-up visits at approximately 3 months (visit 2), 6 months (visit 3), 9 months (visit 4) and 12 months (visit 5). Permissible deviations from scheduled visit dates should not exceed 2 weeks. SAP P11-4- self-assembled peptide, FV-fluoride varnish.

**Group A and B:**

1. Intraoral examination (assessment of malocclusion, parafunctions and dysfunctions);saliva pH and unstimulated salivary flow rate, assessment of BOP, GI and PLI; and dentition assessment using ICDAS-II, ICDAS-LAA, LAA, QLF: Δ*F*, Δ*Q* and lesion area of study teeth after removal of soft and/or hard dental deposits (calculus).
2. Preparation of the tooth surfaces prior to Curodont Repair application:

Prior to application, the treatment surface will be conditioned by removing the pellicle with a 2% sodium hypochlorite solution, followed by rinsing and drying. The WSL will then be etched with 35% phosphoric acid for 20 s, thoroughly rinsed, and dried. The treatment area will be kept under basic isolation using cheek retractors (OptraGate) and cotton rolls.

3. Application of Curodont Repair to the teeth:

The Curodont Repair applicator will be prepared according to the manufacturer's instructions (activation of the applicator to saturate the sponge tip). The saturated applicator will then be applied to the WSL by gentle compression of the sponge tip to deliver the product onto the lesion surface. The material will be left in contact with the lesion for approximately 5 min, during which the participant will be instructed not to rinse.

4. Assessment of AEs during and immediately after the Curodont Repair application, including VAS assessment.
5. Five minutes later: Application of Duraphat fluoride varnish (FV) to all teeth (including the treated teeth). Following moisture control (removal of excess saliva from the working area), an appropriate amount of Duraphat FV will be dispensed onto a dosing pad and applied to the teeth using a standard applicator (brush). The varnish will be distributed in a thin, even film, treating the dentition by quadrants until the required surfaces have been covered. The varnish layer will be left undisturbed and will not be removed immediately after application.
6. Assessment of AEs and VAS after application of CR + FV.

**Group C:**

1. Intraoral examination (assessment of malocclusion, parafunctions and dysfunctions); saliva pH and unstimulated salivary flow rate, assessment of BOP, GI and PLI; and dentition assessment using ICDAS-II, ICDAS-LAA, LAA, QLF: Δ*F*(%), Δ*Q* and lesion area of study teeth after removal of soft and/or hard dental deposits (calculus).
2. Application of Duraphat fluoride varnish (FV) to all teeth (including the treated teeth): Following moisture control (removal of excess saliva from the working area), an appropriate amount of Duraphat fluoride varnish will be dispensed onto a dosing pad and applied to the teeth using a standard applicator (brush). The varnish will be distributed in a thin, even film, treating the dentition by quadrants until the required surfaces have been covered. The varnish layer will be left undisturbed and will not be removed immediately after application.
3. Assessment of adverse events during and immediately after the FV application, including VAS assessment.

#### 2.6.6 Follow-up visits (V2–V5)

Follow-up visits are scheduled at 3 (V2), 6 (V3), 9 (V4), and 12 months (V5).

Groups A and C follow the same procedures at all follow-up visits. Group B additionally receives repeated SAP P11-4 applications at V2; at V3, V4 and V5 Group B follows the same procedures as Groups A and C.

All clinical assessments and QLF imaging will be repeated before application of remineralization agents. Late AEs since the prior visit will be elicited by interview and documented. Prior to clinical and QLF assessment at each follow-up visit, and following the previous intervention and use of remineralization products, soft and/or hard dental deposits will be removed.

**Group A and C:**

Follow-up visits V2-V5.

1. Application of Duraphat fluoride varnish (FV) to all teeth (including the treated teeth): Following moisture control (removal of excess saliva from the working area), an appropriate amount of Duraphat fluoride varnish will be dispensed onto a dosing pad and applied to the teeth using a standard applicator (brush). The varnish will be distributed in a thin, even film, treating the dentition by quadrants until the required surfaces have been covered. The varnish layer will be left undisturbed and will not be removed immediately after application.
2. Assessment of AEs during and immediately after the FV application, including VAS assessment.

**Group B:**

Follow-up visits V2:

1. Preparation of the treated tooth surfaces prior to Curodont Repair application: Prior to application, the treatment surface will be conditioned by removing the pellicle with a 2% sodium hypochlorite solution, followed by rinsing and drying. The WSL will then be etched with 35% phosphoric acid for 20 s, thoroughly rinsed, and dried. The treatment area will be kept under basic isolation using cheek retractors (OptraGate) and cotton rolls.
2. Application of Curodont Repair to the treated teeth: The Curodont Repair applicator will be prepared according to the manufacturer's instructions (activation of the applicator to saturate the sponge tip). The saturated applicator will then be applied to the treated lesion by gentle compression of the sponge tip to deliver the product onto the lesion surface. The material will be left in contact with the lesion for approximately 5 min, during which the participant will be instructed not to rinse.
3. Assessment of AEs during and immediately after the Curodont Repair application, including VAS assessment.
4. Five minutes later: Application of Duraphat fluoride varnish (FV) to all teeth (including the treated teeth). Following moisture control (removal of excess saliva from the working area), an appropriate amount of Duraphat fluoride varnish will be dispensed onto a dosing pad and applied to the teeth using a standard applicator (brush). The varnish will be distributed in a thin, even film, treating the dentition by quadrants until the required surfaces have been covered. The varnish layer will be left undisturbed and will not be removed immediately after application.
5. Assessment of AEs and VAS after application of FV + CR.

Follow-up visits V3–V5:

1. Application of Duraphat fluoride varnish (FV) to all teeth (including the treated teeth): Following moisture control (removal of excess saliva from the working area), an appropriate amount of Duraphat fluoride varnish will be dispensed onto a dosing pad and applied to the teeth using a standard applicator (brush). The varnish will be distributed in a thin, even film, treating the dentition by quadrants until the required surfaces have been covered. The varnish layer will be left undisturbed and will not be removed immediately after application.
2. Assessment of AEs during and immediately after the FV application, including VAS assessment.

#### 2.6.7 Post-treatment instructions

**After FV application**: Participants should be advised not to brush their teeth or chew hard food for at least 4 h after treatment. During this time fluids and soft foods may be consumed.

**After CR application**: Participants could not rinse, eat or drink for 30 min.

**After CR** **+** **FV application**: Participants should be instructed not to eat, drink, or rinse for 30 min. For the following 4 h, they should not brush their teeth or chew hard foods; after 30 min, liquids and soft foods may be consumed.

The characteristics of the investigational and ancillary study products are presented in Table 1.

**Table 1:** Study products.

Product name	Curodont repair	Duraphat fluoride varnish colgate, 22,600 ppm	CHLORAXID 2%	GLUMA Etch Gel	Paste cleanpolish
Manufacturer	vVARDIS (Switzerland)	Colgate-Palmolive (USA)	Cerkamed (Poland)	Kulzer GmbH (Germany)	Kerr (Italy)
Composition	Oligopeptide-104, chlorhexidine digluconate,tromethamine,trehalose dihydrate, hydroxypropylmethylcellulose.	5% sodium fluoride, ethanol 96%, white wax (E901), shellac (E904), colophony, mastic, saccharin (E954), raspberry flavouring (ethyl butyrate, geraniol, iris resinoid, isoamyl acetate, jasmine absolute, propylene glycol, vanillin	Sodium hypochlorite (2% active chlorine)	35% phosphoric acid. Thickening agents; Pigments; Water.	Calcium carbonate, pumice, aluminum oxide.
Storage Conditions	Store at room temperature (the exact storage instructions for the specific batch should always be checked on the product packaging);protected from direct sunlight and heat sources.	Store below 25 °C.	Store sealed in the original packaging; protect from sunlight; store at 2–8 °C (refrigerated) in an upright position.	No special storage requirements; keep containers tightly sealed.	Store at ambient temperature in a dry, well-ventilated area; store separated from acids
Shelf life	See expiry date on the original packaging/label	Unopened: 3 years; after opening: use within 3 months.	Expiry date is on the main container; once opened, the expiry date does not change if the container is securely closed after each use.	See expiry date on the original packaging/label	See expiry date on the original packaging/label

#### 2.6.8 Premature study termination

If, in the Investigator's judgement, clinical observations during the study indicate that continuing the trial may not be advisable, the study may be discontinued at this site in whole or in part. The Investigator will submit a written statement to the Bioethics Committee fully documenting the reasons for early termination.

#### 2.6.9 Duration

The participation time for a single participant is approximately 12 months and includes:

1. Enrollment and Randomization (V0). Following patient eligibility confirmation and the initial assessment, the baseline visit should take place within ± 14 days;
2. The baseline visit (V1);
3. A follow-up visit at approximately 3 months ± 14 days (V2);
4. A follow-up visit at approximately 6 months ± 14 days (V3);
5. A follow-up visit at approximately 9 months ± 14 days (V4);
6. The final visit at approximately 12 months ± 14 days (V5).

### 2.7 Trial staff training and calibration

The study will be conducted at the University Dental Center of the Medical University of Warsaw, Department of Pediatric Dentistry.

Three investigators will be involved in the project.

Two investigators will be trained examiners in the use of the ICDAS-II, ICDAS-LAA, LAA. The training will be conducted by a dentist specialized in pediatric dentistry, with qualifications in the use of ICDAS-II, ICDAS-LAA, LAA. Following the training, intra- and inter-examiners reliability will be evaluated (detection of carious WSL and ICDAS-II and LAA, ICDAS-LAA scoring) on 10% planned number of patients not participating in the study, at the Department of Pediatric Dentistry.

Training in periodontal indices (BOP, PLI and GI) will be provided by a dentist specialized in periodontology and oral mucosal diseases. After this training, intra- and inter-examiners reliability will be assessed (BOP, PLI and GI indices) on 10% planned patients not participating in the study, at the Department of Pediatric Dentistry.

Two investigators will be trained in the application of the enamel remineralization agents in accordance with the manufacturer's recommendations. One investigator will be trained in the use of the QLF camera, QrayCam Pro (Inspektor Research Systems B.V.) through a training session organized by the manufacturer. Three investigators will receive information on possible adverse events and the procedures to be followed if they occur.

### 2.8 Evaluation of carious WSL

#### 2.8.1 Clinical and visual-tactile assessment using ICDAS II

The International Caries Detection and Assessment System II (ICDAS II) is a clinical scoring system used for the detection and assessment of dental caries (18).

In the present study, remineralization agents will be applied to carious lesions scored as Code 1 or 2:

- **Code 0**—Sound enamel
- **Code 1**—Opaque (matte) white or brown spot visible after air-drying
- **Code 2**—Opaque (matte) white or brown spot visible on a wet surface and still visible after air-drying
- **Code 3—**Localized enamel breakdown (no visible dentin): Microcavitation or enamel breakdown due to caries, without visible dentin.
- **Code 4—**Underlying dark shadow from dentin: Shadow of discolored dentin visible through apparently intact enamel, with or without localized enamel breakdown.
- **Code 5—**Distinct cavity with visible dentin: Frank cavitation exposing dentin.
- **Code 6—**Extensive distinct cavity with visible dentin: Obvious, deep and wide cavitation with visible dentin (extensive destruction).

#### 2.8.2 ICDAS-lesion activity assessment (ICDAS-LAA) (Ekstrand criteria)

Lesion activity of the treated WSL will be assessed clinically at each visit using a standardized ICDAS-Lesion Activity Assessment (ICDAS-LAA). The treated WSLs will be assessed on the basis of the lesion's visual appearance (ICDAS II scores), its location with respect to a potential plaque stagnation area and the tactile sensation of the lesion when a periodontal probe is gently run over the lesion after removal of dental plaque.

The description of the ICDAS-LAA criteria and the evaluation of lesion activity are presented in Supplementary Table S4 (19).

#### 2.8.3 Lesion activity assessment (LAA) (Nyvad et al. classification)

The Nyvad classification is a visual-tactile system designed to assess both the activity and severity of carious lesions. The criteria underlying this classification cover the full spectrum of caries development, from clinically sound surfaces, through noncavitated and microcavitated enamel lesions, to obvious cavitation extending into dentin.

The description of the Nyvad et al. criteria for caries lesion activity and severity assessment is presented in Supplementary Table S5 (20).

#### 2.8.4 Quantitative parameter assessment (quantitative light-induced fluorescence; QLF)

At each study visit, QLF measurements will be performed using QLF (Qraycam Pro, Inspektor NL, Amsterdam, The Netherlands). Standardized QLF images will be acquired for the study teeth presenting carious WSL. A dedicated software program, Q-Ray™ Clinical Suite, will be used for this purpose. For image acquisition, an OptraGate (Ivoclar, Schaan, Liechtenstein) retractor will be placed to facilitate adequate lip and cheek retraction and to ensure unobstructed visualization of the dentition.

Participants will be positioned with the dental chair reclined. During imaging, the operator will ensure that the entire dentition is captured without distortion. To minimize reflections, glare and shadowing, a consistent working distance and camera orientation will be maintained; framing will be standardized by centering the dentition in the field of view.

For QLF quantification, a silicone positioning key will be used to reproduce the camera position for each treated lesion across repeated *in vivo* measurements. As fluorescence readings are affected by ambient light and moisture, imaging will be performed under controlled lighting conditions (darkened room) after drying the tooth surface with gauze for approximately 5 s (5). All QLF recordings will be obtained by the same trained investigator using the positioning key to maximize repeatability, in line with previously described methodology.

In addition, alignment between quantitative QLF measurements (Δ*F* values) to ICDAS II scores will be assessed in accordance with Manal Allammari et al. Index (2010) (21) (Supplementary Table S6).

At enrollment and randomization visit (V0) one investigator will assess fluorescence loss (Δ*F*) in the WSLs selected for the clinical study in order to determine whether the lesion is eligible for inclusion.

At the baseline visit (V1), prior to initiation of the application, investigator quantitatively assess:

- **Fluorescence loss Δ*F* baseline (%)**: Percentage reduction in fluorescence relative to sound tooth tissue, reflecting lesion depth. A higher negative number indicates a deeper lesion.
- **Lesion volume Δ*Q* baseline (%** **×** **mm**^2^**)**: Lesion extent combined with its severity (i.e., the degree of tissue demineralization). More negative Δ*Q* values indicate a larger or more severe lesion.
- **Lesion area baseline**: Description of the lesion extent on the tooth surface (mm^2^).

At follow-up visits (V2–V5):

- Fluorescence loss Δ*F follow-up* (%),
- Lesion volume Δ*Q follow-up* (% × mm^2^),
- Lesion area *follow-up*

### 2.9 Evaluation of periodontal parameters

#### 2.9.1 Bleeding on probing (BOP)

BOP will be assessed as an indicator of gingival inflammation. Using a periodontal probe WHO 621 and gentle probing force, the gingival sulcus will be probed at six sites per tooth (mesiobuccal, midbuccal, distobuccal, mesiolingual/palatal, midlingual/palatal, distolingual/palatal) at all teeth. Bleeding will be recorded after probing as present (1) or absent (0) at each site.

The BOP percentage (BOP%) will be calculated as:

BOP%=(Numberofbleedingsites÷Totalnumberofsitesexamined)×100

A patient's gingival status can be categorized as follows: healthy/no gingivitis (BOP% <10%), localized gingivitis (BOP% 10%–30%), or generalized gingivitis (BOP% >30%) (22).

#### 2.9.2 Gingival index (GI)

The Gingival Index (GI) will be used to assess the severity of gingival inflammation at the gingival margin of all teeth at four surfaces per tooth (mesial, distal, buccal, and lingual). Each tooth surface will be scored as:

0 = Normal gingiva, no inflammation
1 = Mild inflammation (slight change in color/edema), no bleeding on probing
2 = Moderate inflammation (redness, edema, glazing), bleeding on probing
3 = Severe inflammation (marked redness/edema, ulceration), spontaneous bleeding or tendency to bleed

The GI for each tooth is calculated by adding the scores from the four areas and dividing by four. The GI for each participant is then calculated by adding the scores for all examined teeth and dividing by the number of teeth examined (23).

- Mild inflammation: 0.1–1.0;
- Moderate inflammation:1.1–2.0;
- Severe inflammation:2.1–3.0

#### 2.9.3 Plaque index (PLI)

The Plaque Index (PLI) will be used to quantify dental plaque accumulation of all teeth at four surfaces per tooth (mesial, distal, buccal, and lingual). Each scored surface will be recorded as:

0 = No plaque
1 = A thin plaque film at the gingival margin detectable by probe or after disclosing
2 = Moderate accumulation of visible plaque at the gingival margin
3 = Abundance of soft deposits covering the tooth surface and/or gingival margin

The PLI for each tooth is calculated by adding the scores from the four areas and dividing by four. The PLI for each participant is then calculated by adding the scores for all examined teeth and dividing by the number of teeth examined (23).

– PLI = 0—The gingival area is literally free of plaque.
– PLI = 1—Gingival area is covered with a thin film of plaque which is not visible
– PLI = 2—The deposit is visible *in situ*
– PLI=3—Heavy (1–2 mm. thick) accumulation of soft matter.

### 2.10 Saliva parameters

#### 2.10.1 Unstimulated whole salivary flow rate

Unstimulated whole saliva will be collected in the morning using a standardized spitting method. Participants will be instructed to refrain from eating, drinking, and smoking for at least 1 h before saliva collection. The patients will be instructed not to brush their teeth or use a mouthwash for at least one hour prior to the scheduled appointment time. Before the measurement, participants will rest for 5 min and swallow all saliva present in the mouth. During collection, participants will be seated with the head tilted forward and instructed to spit all saliva produced into a sterile graduated tube. Saliva will be collected for 5 min, and the unstimulated whole salivary flow rate (UWSFR) will be calculated by dividing the collected volume by the collection time and expressed in mL/min (24).

- **<0.1 mL/min** — Very low salivary flow
- **0.1–0.19 mL/min** — Low salivary flow
- **≥0.2 mL/min** — Normal unstimulated salivary flow

#### 2.10.2 pH of saliva

After collection of unstimulated saliva, a pH indicator strip will be immersed in the saliva sample to measure salivary pH [GC Saliva Check Buffer kits (GC, Tokyo, Japan)]. The result will be read by comparing the colour change of the strip with the manufacturer's reference scale.

PH values above 6.8 correspond to healthy saliva, whereas values between 6.6 and 6 were characterized as moderately acidic, and values below 6 as highly acidic.

### 2.11 Subject withdrawal criteria

Subjects may be withdrawn from the study prematurely for any of the following reasons:

**Intolerable AE:** If a participant experiences an AE that is not tolerated, the Investigator and/or the participant may decide to discontinue participation, irrespective of whether the AE is considered related to the study interventions.
**Intercurrent illness/medical condition:** If a participant develops a concurrent disease or condition that, in the Investigator's judgement, makes continued participation inappropriate.
**Major protocol deviation:** Major deviations that may warrant discontinuation include deviations that could compromise participant safety (e.g., illness requiring treatment), interfere with the assigned intervention, or indicate unwillingness or inability to comply with study procedures and/or oral hygiene instructions. Minor deviations should be corrected when feasible and the participant may continue in the study. All deviations will be documented and assessed for their potential impact on study objectives.
**Non-compliance/non-attendance:** If a participant is uncooperative or repeatedly fails to attend scheduled visits. The Investigator should make reasonable efforts to determine the reason and to facilitate continued attendance.
**Withdrawal of consent:** If the participant (or legal guardian, as applicable) chooses to discontinue participation at any time.
**Pregnancy:** If a female participant becomes pregnant during the study.
All cases of premature withdrawal and their reasons will be reviewed by the Principal Investigator, who will make the final decision regarding study discontinuation.

### 2.12 Safety assessment

All AEs will be recorded from the baseline through the final visit. At each visit, AEs will be elicited by open questioning (e.g., “How have you been feeling since your last visit?”) and by direct inquiry about oral symptoms.

#### 2.12.1 Definition of adverse event

An AE is any unfavorable medical occurrence in a participant who has received a study product, regardless of whether it is considered causally related to the treatment. In this trial, only AEs related to oral health and to the application of the enamel remineralization agents will be recorded and evaluated.

#### 2.12.2 Definition of serious adverse event

A serious adverse event is any untoward medical occurrence that, at any dose/exposure:

1. Results in death;
2. Is life-threatening;
3. Results in persistent or significant disability/incapacity;
4. Requires inpatient hospitalization or prolongation of existing hospitalization;
5. Constitutes an important medical event (i.e., an event that may not be immediately life-threatening and may not result in death or hospitalization, but which, based on appropriate medical and scientific judgement, could jeopardize the participant or may require medical or surgical intervention to prevent one of the serious outcomes listed above).

Hospitalization for elective treatment of a pre-existing condition that has not worsened compared with the baseline is not considered an AE. Likewise, planned hospitalization for a pre-existing condition, or a protocol-required procedure without clinically significant deterioration in health, is not classified as an SAE. All AEs that do not meet any of the criteria above will be considered non-serious. Medical and scientific judgement should be applied when determining whether an event should be classified as serious.

The adverse events associated with the use of the preparations during the study will be assessed based on the presence and frequency of adverse events, the type of reported symptoms [transient tooth hypersensitivity to thermal stimuli, an unpleasant taste sensation, local irritation of the oral mucosa (itching, burning, swelling), a sensation of enamel surface roughness immediately after application, allergic reactions (very rare), and severity (mild, moderate, severe). Possible adverse events will initially be assessed dichotomously as present/absent.

If adverse events occur, they will be assessed as follows:

1. Transient tooth hypersensitivity to thermal stimuli (cold, heat) immediately after the procedure or from several hours up to 1–2 days post-procedure, defined as sharp, short-lasting pain caused by enamel etching prior to the preparation application procedure, recorded on the day of application in the dental office and in the assessment diary;
2. An unpleasant taste sensation due to a slightly bitter or chemical aftertaste of the preparation (after application, the preparation mixes with saliva, which may temporarily intensify the taste sensation) immediately after the procedure, recorded on the day of application in the dental office and in the assessment diary. The unpleasant taste sensation is transient and lasts from several/several dozen minutes up to one hour;
3. Local irritation of the oral mucosa (burning, pain, swelling) immediately after the procedure up to several hours/1–2 days, recorded on the day of application in the dental office and in the assessment diary;
4. Sensation of enamel surface roughness immediately after application, recorded on the day of application in the dental office;
5. Allergic reactions (urticaria, angioedema, anaphylactic shock). Urticaria may present as pruritic, erythematous wheals on the mucosa, appearing suddenly and usually resolving rapidly without residual lesions. Angioedema—deeper swelling of the mucosa, most often of the tongue or pharynx; it may impair breathing. Anaphylactic shock—a severe, life-threatening allergic reaction; symptoms include, inter alia, dyspnea, hypotension, dizziness, tachycardia, and loss of consciousness; it requires immediate medical assistance.

Allergic reactions will be assessed clinically as:

- Mild—local, transient, short-lasting symptoms during or on the day of the procedure (e.g., pruritus, erythema, burning, swelling); no treatment required, observation indicated;
- Moderate—intensified local symptoms requiring pharmacological treatment;
- Severe—allergic reactions (angioedema, anaphylactic shock) requiring immediate medical intervention.

For subjective symptoms, a visual analogue scale (VAS) 0–10 will be used for all symptoms (Supplementary Figure S1).

Adverse events will be recorded in all study participants from the Baseline visit (V1) until the final visit at 12 months (V5). In addition, participants/guardians will have a further 2-week period after study completion to report any AEs.

Between scheduled visits, participants will complete the assessment diary only if an AEs occurs. They will be instructed to record the exact date and time of onset and to describe the symptoms in detail. For participants aged <16 years, the legal guardian will complete the diary entry describing the symptoms. The diary must be brought to each scheduled visit for review.

Unscheduled AEs reporting: Participants/guardians will be provided with the Principal Investigator's email address (available for contact at any time) and telephone number (telephone contact available from 8:00 to 18:00). In the event of severe symptoms, participants/guardians will be instructed to attend the emergency department (ED) or call emergency medical services immediately. For moderate and severe symptoms, participants/guardians will be instructed to notify the study team by email or telephone without delay. In such cases, the Investigator will arrange an unscheduled visit to assess the AEs and associated symptoms.

All AEs will be assessed for severity and for their relationship to the application of the remineralization agents. AEs will be followed until resolution and/or stabilization.

### 2.13 Subject retention

Participant retention will be supported primarily through motivation and education of the participant and the legal guardian. During recruitment and scheduled visits, particular attention will be paid to the presence of carious white spot lesions. Before study initiation, it will be clearly explained what carious white spot lesions are. Using the QLF camera, the participant and the parent/legal guardian will be able to visualize the exact location of the carious white spot lesions, which may enhance engagement in both the treatment and study participation. The participant and guardian will also be informed that, if left untreated, white spot lesions may progress to enamel breakdown and cavitated caries, potentially leading to deterioration of oral health and compromised aesthetics in the anterior dentition. In addition, participants will be informed that the study-related treatment is provided free of charge, meaning they will not incur any costs for the intervention.

### 2.14 Statistical analysis

Analyses will be conducted using R (R Foundation for Statistical Computing, Vienna, Austria) or Python (with validated statistical libraries such as statsmodels and/or PyMC). Baseline characteristics will be summarized at the patient level by treatment group. Continuous variables will be presented as mean ± standard deviation or median (interquartile range), as appropriate, while categorical variables will be summarized as counts and percentages. The primary outcome is the change in the QLF-derived Δ*Q* parameter from baseline to the 6-month follow-up (prespecified primary time point). The primary comparison is the between-group difference in this change between the repeated-application and single-application SAP P11-4 groups, modeled via linear mixed-effects models with fixed effects for treatment group, time, and their interaction, and random intercepts for patient and lesion. Δ*F* will be analyzed separately as a key secondary QLF outcome, using an analogous modeling approach. The primary comparison will be tested at two-sided *α* = 0.05 without further multiplicity adjustment. Where multiple pairwise comparisons are performed within the same outcome and time point (e.g., across the three study groups), multiplicity will be controlled using the Holm procedure. Secondary outcomes will be analyzed according to their measurement scale, without confirmatory claims. Continuous outcomes will be analyzed using LMMs analogous to the primary analysis. Ordinal outcomes will be analyzed using ordinal logistic mixed-effects models, including random effects for patient (and lesion where applicable). These variables will not be treated as continuous unless distributional properties and scale characteristics justify such an approximation. Binary outcomes will be analyzed using mixed-effects logistic regression models.

### 2.15 Protocol compliance and deviations

#### 2.15.1 Major protocol deviations

Major protocol deviations are deviations that may materially affect data integrity, increase participant risk, substantially reduce the expected treatment benefit, or compromise the interpretation of trial results. Major deviations include, but are not limited to:

- Randomization/enrollment of a participant who does not meet inclusion/exclusion criteria at the baseline or at re-confirmation.
- Incorrect treatment allocation or administration of the wrong intervention.
- Missing the baseline assessment of key efficacy variables (e.g., missing the baseline QLF measurement and/or inability to identify/record the treated lesions at the baseline).
- Missing the 6-month assessment of the primary efficacy outcome (Δ*Q*) and/or the final 12-month assessment of key efficacy variables (QLF).
- Use of prohibited concomitant treatments likely to affect enamel remineralization on lesion appearance.
- Major non-compliance with study procedures or oral hygiene instructions that, in the Investigator's judgement, could invalidate endpoint assessment (e.g., repeated failure to attend visits, refusal of required assessments).
- Treated WSL is no longer evaluable due to non-study dental procedures affecting the study surface (e.g., restoration, orthodontic attachment placed on the lesion surface, trauma affecting the surface) when this prevents QLF/clinical assessment.
- Serious safety issue leading to withdrawal.

#### 2.15.2 Minor protocol deviations

Minor protocol deviations are deviations expected to have minimal impact on participant safety or on the quality/interpretability of trial data. Minor deviations include, but are not limited to:

- Visit performed slightly outside the planned visit window.
- Missed secondary assessment at a given visit (e.g., missing GI, PLI or BOP), while primary assessments are completed.
- Partially incomplete questionnaire or missing answers to individual survey items.
- Minor deviations from standardized pre-QLF preparation (small variation in drying time) provided images remain evaluable and meet minimum quality criteria.
- Temporary technical issues (software/camera) causing delays, with successful completion of imaging within the same visit or at the earliest feasible opportunity.
- Self-reported minor non-adherence to post-procedure instructions (e.g., eating earlier than recommended), documented without safety consequences.

All protocol deviations will be documented, reviewed, and assessed for potential impact on the study objectives; corrective actions will be implemented where feasible.

The Principal Investigator is responsible for overseeing adherence to the protocol and for recording all protocol deviations.

1. No planned departures from the protocol (waivers/exemptions) are permitted; for example, participants will not be enrolled if they fail to meet the eligibility criteria or any protocol-defined restrictions.
2. Any unintentional deviation occurring during the study will be documented on the appropriate study records and promptly communicated to the Principal Investigator.
3. Recurrent deviations are considered unacceptable, will trigger immediate corrective action, and may be assessed as a serious breach.

### 2.16 Amendments

If changes to this protocol are necessary:

- The Principal Investigator will authorize any protocol change and will determine whether it constitutes a substantial or non-substantial amendment.
- Substantial amendments will be submitted to the Bioethics Committee of the Medical University of Warsaw for approval prior to implementation.
- An amendment history will be maintained in the protocol appendices. The protocol version and date will be clearly displayed on the title page and in the footer of each page.

### 2.17 Indemnity

Because this is an interventional clinical study, appropriate liability insurance will be obtained prior to study initiation in accordance with applicable regulations.

By signing this protocol, the Principal Investigator confirms agreement with its contents and commits to conducting the study in accordance with the protocol and all applicable regulatory requirements.

### 2.18 Data management

Documentation will be divided into electronic and paper records. Electronic documentation will be stored on a laptop and will be used to archive QLF images and measurements (QLF software, Inspektor NL, The Netherlands). Paper documentation will include participant data, the legal guardian's contact telephone number, information on clinical and visual-tactile and quantitative assessments, adverse events, VAS scores, and visit numbering. All documentation will be stored on the premises of the University Clinical Center of the Medical University of Warsaw, Department of Pediatric Dentistry, in a locked location. Access to the documentation will be restricted exclusively to the study investigators.

## 3 Results

This manuscript describes a study protocol; therefore, no results are currently available. The final results are expected to be obtained 12 months and 2 weeks after completion of the last study visit.

The primary outcome will focus on the change in the QLF-derived Δ*Q* parameter from baseline to 6 months, comparing repeated vs. single SAP P11-4 application. Remineralization in all study groups will be interpreted as an increase in Δ*F* and Δ*Q* values toward zero, indicating reduced fluorescence loss and lesion severity, respectively. In addition to changes in Δ*F* and Δ*Q*, changes in lesion area, ICDAS, LAA, ICDAS-LAA scores, periodontal parameters, and oral hygiene and dietary habits are expected to be assessed.

We expect no adverse events, or only mild adverse events, related to the intervention. The study will assess whether salivary pH and unstimulated salivary flow rate influence the remineralization outcome. In addition, the study will evaluate whether repeated application of SAP P11-4 is effective and provides a durable remineralizing effect over the follow-up period.

## 4 Discussion

The planned study will evaluate the effectiveness of a single application of SAP P11-4 compared with repeated application in the treatment of non-cavitated enamel white spot lesions over a 12-month follow-up period. A major strength of this study is the use of QLF as a quantitative method for monitoring remineralization. QLF parameters will allow assessment of treatment effects from several perspectives, including fluorescence loss, lesion volume, and lesion area. Therefore, the study will not rely only on visual or qualitative assessment, but will combine quantitative outcomes with clinical evaluation. This is consistent with previous clinical studies in which WSLs were assessed using combined clinical and instrumental methods, such as ICDAS, DIAGNOdent or QLF (3, 5, 8, 9, 12, 25, 26).

Several limitations should be considered. First, dietary and hygiene habits may influence the remineralization process, and it is not possible to control patients' diet and hygiene over the 12-month observation period. Similarly, although standardized oral hygiene instructions will be provided to all participants, individual compliance with oral hygiene and dietary recommendations may vary during follow-up. Soliman et al. (25) also emphasized oral hygiene and healthier dietary habits as important part of the non-invasive management of WSLs, which supports the need to standardize these factors as much as possible in the present study.

Second, salivary conditions may affect remineralization outcomes. Remineralization depends on the balance between pathological and protective factors in the oral environment. Calcium and phosphate ions present in saliva participate in mineral redeposition within demineralized tooth structure, while pH changes influence whether demineralization or remineralization predominates (27). Salivary pH and unstimulated salivary flow rate may act as confounding factors, as saliva contributes to acid neutralization and pH recovery in the oral environment, thereby influencing the balance between demineralization and remineralization.

Third, QLF parameters such as Δ*F* and Δ*Q* and lesion area are continuous quantitative measures, and no universally accepted minimal clinically important difference has been established for QLF-derived outcomes in white spot lesions. For this reason, statistically significant changes in QLF parameters will not be interpreted in isolation. The clinical relevance of QLF findings will be confirmed by corresponding changes in clinical outcomes, including lesion area, ICDAS, LAA, and ICDAS-LAA scores. The results of this study may contribute to the development of a more predictable clinical protocol for the non-invasive management of early enamel white spot lesions.

The study findings will be shared through publications in reputable peer-reviewed journals and presentations at scientific conferences. A plain-language summary of the results will be made available on the project website for participants and the general public. The Investigators may publish the results once the trial has been completed. In addition, study outputs and associated datasets will be made accessible to the research community in line with the project's open science requirements.

## 5 Conclusion

This trial will provide robust evidence on the comparative efficacy of single vs. repeated SAP P11-4 application in the management of enamel WSLs, and will contribute to establishing an evidence-based clinical protocol for the use of this biomimetic agent in adolescent patients.

## Data Availability

The original contributions presented in the study are included in the article/Supplementary Material, further inquiries can be directed to the corresponding authors.
