# Supplementary material for: Three-arm randomized controlled trial evaluating the effectiveness of the self-assembling peptide P11-4 in the remineralization of enamel carious white spot lesions: a study protocol
Source: Front Oral Health. 2026 Oct 7;7:1932140. doi: 10.3389/froh.2026.1932140 (PMC13683189; doi:10.3389/froh.2026.1932140)
Supplement: Supplementary file 1 [file Table1.docx]

Supplementary Material

Three-arm Randomized Controlled Trial Evaluating the Effectiveness of the Self-Assembling Peptide P11-4 in the Remineralization of Enamel Carious White Spot Lesions: a Study Protocol

| **Procedure** | **Enrolment**  **(V0)** | **Baseline (V1)** | **3 months**  **(V2)** | **6 months**  **(V3)** | **9 months**  **(V4)** | **12 months**  **(V5)** |
| --- | --- | --- | --- | --- | --- | --- |
| **Eligibility Criteria**  **check** | X | X | X | X | X | X |
| **Study questionnaire** |  | X |  |  |  |  |
| **BOP** |  | X | X | X | X | X |
| **GI** |  | X | X | X | X | X |
| **PLI** |  | X | X | X | X | X |
| **ICDAS II**. | X | X | X | X | X | X |
| **Presence of dental calculus** | X | X | X | X | X | X |
| **Unstimulated saliva and pH saliva** |  | X | X | X | X | X |
| **LAA of the study teeth** | X | X | X | X | X | X |
| **ICDAS-LAA of the study teeth.** | X | X | X | X | X | X |
| **QLF: ΔF (%)** | X | X | X | X | X | X |
| **QLF:ΔQ (%)** |  | X | X | X | X | X |
| **QLF: Lesion area (mm2)** |  | X | X | X | X | X |
| **SAP P11-4 application** |  | X |  |  |  |  |
| **FV application** |  | X | X | X | X | X |
| **Removal of soft plaque deposits** | X | X | X | X | X | X |
| **AE** |  | X | X | X | X | X |

**Table S1. Study Schedule of Procedures (Group A)**

*BOP- bleeding on probing, GI-gingival index, PLI-plaque index, ICDAS II-international caries detection and assessment system, ICDAS-LAA- ICDAS-lesion activity assessment, LAA-lesion activity assessment, QLF-quantitative light induced fluorescence, ΔF- loss of fluorescence, ΔQ-volume, SAP P11-4- self-assembled peptide, FV-fluoride varnish, AE-Assessment of adverse events*

| **Procedure** | **Enrolment**  **(V0)** | **Baseline (V1)** | **3 months**  **(V2)** | **6 months**  **(V3)** | **9 months**  **(V4)** | **12 months**  **(V5)** |
| --- | --- | --- | --- | --- | --- | --- |
| **Eligibility Criteria**  **check** | X | X | X | X | X | X |
| **Study questionnaire** |  | X |  |  |  |  |
| **BOP** |  | X | X | X | X | X |
| **GI** |  | X | X | X | X | X |
| **PLI** |  | X | X | X | X | X |
| **ICDAS II** | X | X | X | X | X | X |
| **Presence of dental calculus** | X | X | X | X | X | X |
| **Unstimulated saliva and pH saliva** |  | X | X | X | X | X |
| **LAA of the study teeth** | X | X | X | X | X | X |
| **ICDAS-LAA of the study teeth.** | X | X | X | X | X | X |
| **QLF: ΔF (%)** | X | X | X | X | X | X |
| **QLF:ΔQ (%)** |  | X | X | X | X | X |
| **QLF: Lesion area (mm2)** |  | X | X | X | X | X |
| **SAP P11-4 application** |  | X | X |  |  |  |
| **FV application** |  | X | X | X | X | X |
| **Removal of soft plaque deposits** | X | X | X | X | X | X |
| **AE** |  | X | X | X | X | X |

**Table S2. Study Schedule of Procedures (Group B)**

*BOP- bleeding on probing, GI-gingival index, PLI-plaque index, ICDAS II-international caries detection and assessment system, ICDAS-LAA- ICDAS-lesion activity assessment, LAA-lesion activity assessment, QLF-quantitative light induced fluorescence, ΔF- loss of fluorescence, ΔQ-volume, SAP P11-4- self-assembled peptide, FV-fluoride varnish, AE-Assessment of adverse events*

| **Procedure** | **Enrolment**  **(V0)** | **Baseline (V1)** | **3 months**  **(V2)** | **6 months**  **(V3)** | **9 months**  **(V4)** | **12 months**  **(V5)** |
| --- | --- | --- | --- | --- | --- | --- |
| **Eligibility Criteria**  **check** | X | X | X | X | X | X |
| **Study questionnaire** |  | X |  |  |  |  |
| **BOP** |  | X | X | X | X | X |
| **GI** |  | X | X | X | X | X |
| **PLI** |  | X | X | X | X | X |
| **ICDAS II** | X | X | X | X | X | X |
| **Presence of dental calculus** | X | X | X | X | X | X |
| **Unstimulated saliva and pH saliva** |  | X | X | X | X | X |
| **LAA of the study teeth** | X | X | X | X | X | X |
| **ICDAS-LAA of the study teeth.** | X | X | X | X | X | X |
| **QLF: ΔF (%)** | X | X | X | X | X | X |
| **QLF:ΔQ (%)** |  | X | X | X | X | X |
| **QLF: Lesion area (mm2)** |  | X | X | X | X | X |
| **SAP P11-4 application** |  |  |  |  |  |  |
| **FV application** |  | X | X | X | X | X |
| **Removal of soft plaque deposits** | X | X | X | X | X | X |
| **AE** |  | X | X | X | X | X |

**Table S3. Study Schedule of Procedures (Group C)**

*BOP- bleeding on probing, GI-gingival index, PLI-plaque index, ICDAS II-international caries detection and assessment system, ICDAS-LAA-lesion activity assessment, LAA-lesion activity assessment, QLF-quantitative light induced fluorescence, ΔF- loss of fluorescence, ΔQ-volume, SAP P11-4- self-assembled peptide, FV-fluoride varnish, AE-Assessment of adverse events*

| **Clinical parameter** | **ICDAS score** | **Points** |
| --- | --- | --- |
| Clinical parameter 1 (visual appearance: severity score) | ICDAS score 1,2 (brown lesions) | 1 point |
|  | ICDAS score 1,2 (white lesions) | 3 points |
|  | ICDAS score 3, 4, 5 or 6 | 4 points |
| Clinical parameter 2 (gingival inflammation with plaque  stagnation) | Gingival inflammation with plaque stagnation | 3 points |
|  | No gingival inflammation | 1 point |
| Clinical parameter 3 (surface texture) | Rough or soft surface on gentle probing | 4 points |
|  | Smooth or hard surface on gentle probing | 2 points |
|  |  |  |
| Final sum | ≤7 | Inactive caries |
|  | >7 | Active caries |

**Table S4. ICDAS-Lesion activity assessment (ICDAS-LAA) criteria according to Ekstrand et al. [19]**

| **Score** | **Category** | **Criteria** |
| --- | --- | --- |
| 0 | Sound | Normal enamel translucency and texture (slight staining allowed in otherwise sound fissure) |
| 1 | Active caries  (intact surface) | Surface of enamel is whitish/yellowish opaque with loss of luster; feels rough when the tip of the probe is moved gently across the surface; generally covered with plaque  No clinically detectable loss of substance  Smooth surface: caries lesion typically located close to gingival margin  Fissure/pit: intact fissure morphology; lesion extending along the walls of the fissure |
| 2 | Active caries  (surface discontinuity) | Same criteria as score 1  Localized surface defect (microcavity) in enamel only  No undermined enamel or softened floor detectable with the explorer |
| 3 | Active caries  (cavity) | Enamel/dentin cavity easily visible with the naked eye; surface of cavity feels soft or leathery on  gentle probing  There may or may not be pulpal involvement |
| 4 | Inactive caries  (intact surface) | Surface of enamel is whitish, brownish or black  Enamel may be shiny and feels hard and smooth when the tip of the probe is moved gently across  the surface  No clinically detectable loss of substance  Smooth surface: caries lesion typically located at some distance from gingival margin  Fissure/pit: intact fissure morphology; lesion extending along the walls of the fissure |
| 5 | Inactive caries  (surface discontinuity) | Same criteria as score 4  Localized surface defect (microcavity) in enamel only  No undermined enamel or softened floor detectable with the explorer |
| 6 | Inactive caries  (cavity) | Enamel/dentin cavity easily visible with the naked eye; surface of cavity may be shiny and feels  hard on probing with gentle pressure  No pulpal involvement |
| 7 | Filling (sound surface) |  |
| 8 | Filling + active caries | Caries lesion may be cavitated or noncavitated |
| 9 | Filling + inactive caries | Caries lesion may be cavitated or noncavitated |

**Table S5. Description of the Nyvad criteria for caries lesion activity and severity assessment (LAA) [Nyvad et al., 1999] [20]**

| **QLF**  **SCORE** | **ICDAS**  **SCORE** | **DESCRIPTION** | **∆F** |
| --- | --- | --- | --- |
| **0** | **0** | **Sound tooth surface** | **[-0,5 to -12]** |
| **1** | **1** | **First visual change in enamel** | **[-12,5 to -18]** |
| **2** | **2** | **Distinct visual change in enamel** | **[-18,5 to -26]** |
| **3** | **3** | **Localized enamel breakdown**  **because of caries with no visible**  **dentin or underlying shadow** | **[-26,5 to -32]** |
|  | **4** | **Underlying dark shadow from**  **dentin with or without localized**  **enamel breakdown** | **[-32,5 to -38]** |
| **4** | **5** | **Distinct cavity with visible dentin** | **[-38,5 to -48]** |
|  | **6** | **Extensive distinct cavity with**  **visible dentin** | **[>- 48,5]** |

**Table S6. Manal Allammari et al. Index (2010). Ranges of ΔF values corresponding to ICDAS scores [21]**

**Figure S1.** Visual Analogue Scale (VAS) used for patient-reported tolerability assessment.
